# Comparative cytogenetics of three species of *Dichotomius* (Coleoptera, Scarabaeidae)

**DOI:** 10.1590/S1415-47572009005000040

**Published:** 2009-05-10

**Authors:** Guilherme Messias da Silva, Edgar Guimarães Bione, Diogo Cavalcanti Cabral-de-Mello, Rita de Cássia de Moura, Zilá Luz Paulino Simões, Maria José de Souza

**Affiliations:** 1 Centro de Ciências Biológicas, Departamento de Genética, Universidade Federal de Pernambuco, Recife, PEBrazil; 2 Departamento de Biologia, Faculdade de Filosofia, Ciências e Letras de Ribeirão Preto, Universidade de São Paulo, Ribeirão Preto, SPBrazil; 3 Instituto de Ciências Biológicas, Departamento de Biologia, Universidade de Pernambuco, Recife, PEBrazil

**Keywords:** chromosome rearrangements, heterochromatin, karyotype, NORs, FISH

## Abstract

Meiotic and mitotic chromosomes of *Dichotomius nisus*, *D. semisquamosus* and *D. sericeus* were analyzed after conventional staining, C-banding and silver nitrate staining. In addition, *Dichotomius nisus* and *D. semisquamosus* chromosomes were also analyzed after fluorescent *in situ* hybridization (FISH) with an rDNA probe. The species analyzed had an asymmetrical karyotype with 2n = 18 and meta-submetacentric chromosomes. The sex determination mechanism was of the Xy_p_ type in *D. nisus* and *D. semisquamosus* and of the Xy _r_ type in *D. sericeus*. C-banding revealed the presence of pericentromeric blocks of constitutive heterochromatin (CH) in all the chromosomes of the three species. After silver staining, the nucleolar organizer regions (NORs) were located in autosomes of *D. semisquamosus* and *D. sericeus* and in the sexual bivalent of *D. nisus*. FISH with an rDNA probe confirmed NORs location in *D. semisquamosus* and in *D. nisus*. Our results suggest that chromosome inversions and fusions occurred during the evolution of the group.

## Introduction

The family Scarabaeidae belongs to the suborder Polyphaga and has approximately 2,000 genera and 25,000 species (Costa, 2000). This family reunites the majority of the New World coleopterans, with 362 genera and 4,706 species in the Neotropical area and about 125 genera and 1,700 species in the Nearctic region (Costa, 2000; Ratcliffe *et al.*, 2002). In Brazil, approximately 204 genera and 1,777 species have been recognized (Costa, 2000). The tribe Coprini presents about 30 genera, including *Dichotomius*, with more than 750 widely distributed species, from which over 600 occur in the New World and 83 were reported in Brazil (Vaz-de-Mello, 2000; Ratcliffe *et al.*, 2002).

In spite of the large number of Scarabaeidae species described cytogenetic analyses are still scarce in this group and predominantly restricted to standard analysis. Cytogenetic data are available for around 390 scarabaeids which corresponds to approximately 1,5% of the described species (Smith and Virkki, 1978; Yadav *et al.*, 1979; Vidal, 1984; Moura *et al.*, 2003; Bione *et al.*, 2005a, 2005b; Wilson and Angus, 2005; Angus *et al.*, 2007; Dutrillaux *et al.*, 2007; Cabral-de-Mello *et al.*, 2008). Most Scarabaeidae species presented a karyotype with 2n = 20 with meta-submetacentric chromosomes and a parachute sex chromosome mechanism (Xy_p_), but the diploid numbers ranged from 2n = 8 to 2n = 30 and seven different sex determination mechanisms have been reported (Smith and Virkki, 1978; Yadav *et al.*, 1979; Vidal, 1984; Martins, 1994; Colomba *et al.*, 1996, 2000a; Cabral-de-Mello *et al.*, 2007).

Only some of the Scarabaeidae species had their chromosomes analyzed after banding: 65 species had their chromosomes analyzed after C-banding, base-specific fluorochromes were used in ten species, 14 species were studied after silver staining and 11 were analyzed after fluorescent in situ hybridization (FISH) with a ribosomal DNA (rDNA) probe. In general, species from this family presented constitutive heterochromatin (CH) in the pericentromeric areas of the autosomes and some species showed additional heterochromatin in interstitial and/or telomeric areas (Moura *et al.*, 2003; Bione *et al.*, 2005a, 2005b; Dutrillaux *et al.*, 2007). Five types of distribution were reported for the nucleolar organizer regions (NORs): 1) NORs located in one autosomal pair, in *Lygerus ebenus* and *Phyllophaga* (*Phyllophaga*) aff *capillata*; 2) NORs present in more than one autosomal pair, in *Gymnopleurus sturmi* and *Bubas bison*; 3) NORs in many autosomes and in the X chromosome, in *Diabroctisminas*; 4) NORs restricted to the X chromosome, in *Phyllophaga* (*Phytalus*) *vestita*, *Lyogenys fuscus*, *Geniates borelli*, *Macraspis festiva*, *Pelidnota pallidipennis* and *Pentodon bidens punctatum*; and 5) NORs in the X and Y chromosomes, in *Jumnos ruckeri* (Colomba *et al.*, 2000a; Moura *et al.*, 2003; Vitturi *et al.*, 2003; Bione *et al.*, 2005a, 2005b; Mascaine *et al.*, 2007).

The aim of this study was to describe and compare the karyotypes of *Dichotomius nisus*, *D. semisquamosus* and *D. sericeus* (Scarabaeinae, Coprini), using C-banding, silver nitrate staining and FISH with a 28S rDNA probe.

**Figure 1:**
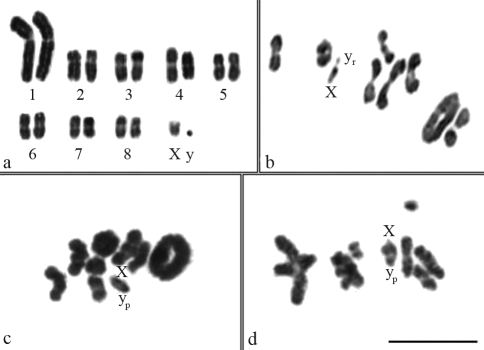
Meiotic and mitotic cells of *Dichotomius sericeus* (a, b), *D. nisus* (c) and *D. semisquamosus* (d): (a) spermatogonial karyotype, 2n = 18; (b) metaphase I showing the Xy_r_ mechanism, n = 8II+Xy_r_; (c) metaphase I, n = 8II+Xy_p_; (d) diakinesis with an overlap of two autosomal pairs, n = 8II+Xy_p._ Bar = 10 μm.

**Figure 2:**
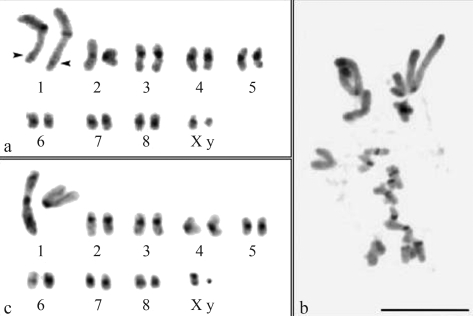
C-banding in spermatogonial metaphases of *Dichotomius nisus* (a), *D. semisquamosus* (b) and *D. sericeus* (c). The arrowheads in (a) indicate the weak interstitial CH block. Bar = 10 μm.

**Figure 3:**
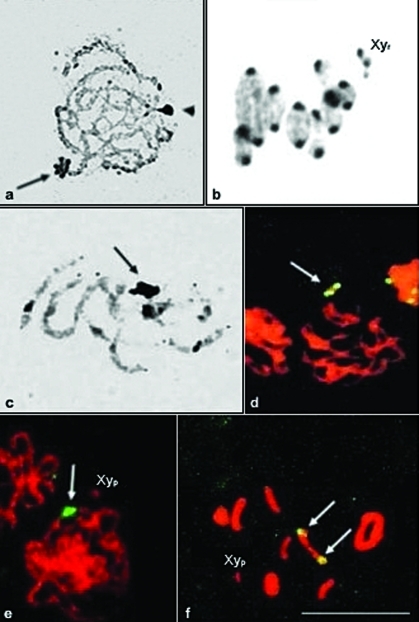
Silver staining of the nucleolar organizer regions (NORs), and of the constitutive heterochromatin and rDNA sites in *Dichotomius sericeus* (a, b), *D. nisus* (c, d) and *D. semisquamosus* (e, f). (a) Pachytene with a totally stained autosomal bivalent (arrow), the arrowhead points to the sexual bivalent; (b) Metaphase I with silver stained heterochromatic segments. Note the Xy_r_ sexual bivalent; (c) Pachytene with a totally stained sexual bivalent (arrow); (d, e) Pachytene and (f) Metaphase I showing the rDNA sites (arrows). Note the Xy_p_ sexual bivalent. Bar = 10 μm.

## Materials and Methods

Meiotic and mitotic chromosomes of adult males of the genus *Dichotomius* Hope, 1838 captured from 2003 to 2006 with the use of “pitfall” soil traps were studied. Fifteen specimens of *Dichotomius nisus* (Olivier, 1789) and 18 of *D. semisquamosus* (Curtis, 1845) were collected at Aldeia, in the district of Paudalho (7° 53' 48" S, 35° 10' 47" W) and in Brejo Novo, district of Caruaru (8° 17' 0" S, 35° 58' 34" W), state of Pernambuco, Brazil and in the city of Ribeirão Preto (21° 10' 39" S, 47° 48' 37" W), São Paulo, Brazil. The 50 specimens of *Dichotomius sericeus* (Harold, 1867) were collected exclusively in the localities in the state of Pernambuco.

The individuals were sacrificed, their testes were fixed in Carnoy (3:1 ethanol:acetic acid) and stored in the freezer at -20 °C. The cytological preparations were obtained using the standard procedure and were stained with 2% lacto-acetic orcein. C-banding was performed according to Sumner (1972) and silver nitrate staining was performed according to Rufas *et al.* (1987). For fluorescent *in situ* hybridization (FISH), the methodology used was that described by Natarajan *et al.* (1998) and Sakamoto-Hojo *et al.* (1999). The rDNA fragment used as probe for NOR detection was obtained from an *Apis mellifera* 28S clone (GenBank access number AJ302936). After polymerase chain reaction (PCR) amplification, the resulting fragments were used as templates in another PCR cycle to obtain biotinylated products. Avidin coupled with fluorescein isothiocyanate (FITC) was used for probe detection. The analyses were performed under a Leica microscope and the CytoVision system coupled to an Olympus BX51 microscope was used for obtaining of the photomicrographs. The figures were organized using the Corel Photo-Paint 12 software.

## Results

*Dichotomius sericeus*, *D. nisus* and *D. semisquamosus* presented 2n = 18 chromosomes. These species had similar karyotypes with meta-submetacentric chromosomes that showed a gradual decrease in size, except for the especially large pair 1 (Figure 1). As for the sex determining system , *Dichotomius sericeus* presented a Xy_r_ mechanism (meioformula 8II+Xy_r_) (Figure 1a, b), while *D. nisus* and *D. semisquamosus* had a Xy_p_ system (meioformula 8II+Xy_p_), with a medium X chromosome and a minute y chromosome, characterizing a typical parachute (Figure 1c, d).

The autosomes of the three species presented blocks of pericentromeric constitutive heterochromatin (CH) (Figure 2). *Dichotomius nisus* showed an additional weak interstitial block of CH in the long arm of pair 1 (Figure 2a). The distribution of CH in *D. semisquamosus* was inconclusive but most of the chromosomes had pericentromeric blocks (Figure 2b). The CH blocks of this species were smaller than those of *D. nisus* and *D. sericeus*. The X chromosome of *D. nisus* presented a CH pericentromeric block, while in *D. sericeus* the CH was located in the short arm of the X chromosome. The y chromosome was heterochromatic in *D. sericeus* and euchromatic in *D. nisus* (Figure 2 a, c).

Silver nitrate staining revealed amorphous masses corresponding to the nucleolar remnants (NORs) in the three species and associated to the sexual bivalent in *D. nisus* (Figure 3c). Analyses of different cells indicated that the NOR is probably associated with an autosome in *D. sericeus* (Figure 3a). The results also indicated that the NOR was present in one medium autosome pair in *D. semisquamosus* (data not shown). The corresponding heterochromatic areas were also stained by silver nitrate in different meiotic phases (Figure 3b). FISH with the 28S rDNA probe confirmed the distribution pattern of rDNA clusters already observed after silver staining in *D. nisus* and *D. semisquamosus* (Figure 3 d-f).

## Discussion

Around half of the species of the subfamily Scarabaeinae cytogenetically analyzed presented variation in diploid number or in the sex determination mechanism (2n = 20, Xy_p_) considered primitive in the family Scarabaeidae and for the order Coleoptera (Smith and Virkki, 1978). Scarabaeinae is the most karyotypically diverse subfamily of Scarabaeidae, with variation in diploid numbers and sex determining mechanisms (Yadav and Pillai, 1979; Martins, 1994; Colomba *et al.*, 1996; Vitturi *et al.*, 2003; Bione *et al.*, 2005a, 2005b, Cabral-de-Mello *et al.*, 2008).

The three species of *Dichotomius* analyzed herein, as well as *D. anaglypticus* (= *D. bos*) (Vidal, 1984) and *D. geminatus* (Cabral-de-Mello *et al.*, 2008) presented a karyotype with 2n = 18 and biarmed chromosomes. The relatively large size of pair 1, which corresponded to the largest element of the complement, characterized a karyotypic asymmetry in these species. The reduction of the diploid number to 2n = 18 and the relatively larger size of pair 1 when compared to the other chromosomes of the karyotype suggests the occurrence of a pericentric inversion followed by a fusion between autosomes from an ancestral karyotype with 2n = 20. Similar rearrangements have already been described and represent the main karyotypic changes involved in the chromosomal evolution of Scarabaeidae (Yadav and Pillai, 1979, Bione *et al.*, 2005a, 2005b; Cabral-de-Mello *et al.*, 2007, 2008). Other species of the genus *Dichotomius*, such as *Pinotus carolinus* (= *Dichotomius carolinus*) and *D. bosqui*, conserved the primitive 2n = 20 karyotype (Smith and Virkki, 1978; Vidal, 1984).

*Dichotomius nisus* and *D. semisquamosus* showed an achiasmatic Xy_p_ sex determination mechanism, considered primitive for Scarabaeidae. This mechanism has also been described in other species of *Dichotomius* (Smith and Virkki, 1978; Yadav *et al.*., 1979; Vidal, 1984, Cabral-de-Mello *et al.*, 2008). *Dichotomius sericeus* presented a Xy_r_ sex determination mechanism, less frequent for Scarabaeidae and only reported in five Scarabaeinae: *Catharsius* sp., *C. molosus*, *Onthophagus bonasus* (= *Diginthophagus bonasus)*, *O. catta* (= *D. gazella*) and *O. dama*; in one Melolonthinae: *Autoserica* sp.; and in four Rutelinae: *Adorrhinyptia* sp., *Anomalous lucens*, *Strigodermella protea* and *Ectinohoplia rufipes* (Smith and Virkki, 1978; Yadav *et al.*, 1979).

*Dichotomius sericeus*, *D. nisus* and *D. semisquamosus* presented pericentromeric constitutive heterochromatin (CH), a pattern also observed in other Coleoptera (Colomba *et al.*, 2000a, 2000b; Moura *et al.*, 2003; Rozek *et al.*, 2004; Bione *et al.*, 2005a, 2005b). Several CH distribution patterns have been described in Scarabaeidae. *Eucranium arachnoids*, for instance, presented pericentromeric C-banded regions and telomeric heterochromatic blocks in pairs 4, 6, 8 and in the X chromosome; no CH was observed in pair 1 and in the Y chromosome (Vidal and Nocera, 1984). In *Bubas bison*, besides the pericentromeric CH, additional distal heterochromatic blocks were reported in eight chromosome pairs (Colomba *et al.*, 1996, 2006). *Isocopris inhiata* and *Diabroctis mimas* possessed large CH blocks, which corresponded to the whole short arms of pairs 3, 4, 5 and 7 in the first species, and to the short arms of pairs 2, 4 and 7 in the latter species. The X chromosomes of these species were almost totally heterochromatic (Bione *et al.*, 2005a).

Silver nitrate staining of CH in *D. nisus*, *D. semisquamosus* and *D. sericeus* was similar to the patterns reported for other species of Scarabaeoidea, as *Bubas bison*, *Pelidnota pallidipenis*, *Dorcus parallelipipedus* and *Thorectes intermedius* (Vitturi *et al.*, 1999; Colomba *et al.*, 2000b, 2006; Bione *et al.*, 2005b). The silver nitrate staining did not depend on the composition of the CH, but was possibly related to proteins associated with these areas.

The absence of silver nitrate staining in the sexual bivalent of *D. sericeus* corroborated the occurrence of the derived Xy_r_ mechanism. In other species with Xy_p_, the lumen of this bivalent was stained by silver due to the presence of argyrophilic proteins. According to Virkki *et al.* (1990; 1991), these proteins possess an adhesive function between the sex chromosomes, controlling their association and correct segregation during meiotic metaphase I and anaphase I, respectively.

The most common NORs distribution pattern reported in Coleoptera is one autosomal pair bearing the nucleolar organizer. In Scarabaeidae, NORs are frequently distributed in a single autosomal pair or in the sexual bivalent (Virkki, 1983; Colomba *et al.*, 2000a; Moura *et al.*, 2003; Vitturi *et al.*, 2003; Bione *et al.*, 2005a, 2005b). Variation in NORs location was observed in the species studied herein. In *Dichotomius semisquamosus* and *D. sericeus*, the NORs were restricted to autosomes. This pattern was also found by Moura *et al.* (2003) in *Phyllophaga* (*Phyllophaga*) aff. *capillata* after silver nitrate staining and FISH. In *D. nisus* the NOR was located in the sexual bivalent, as was also reported for *Phyllophaga* (*Phytalus*) *vestita* and *Lyogenys fuscus* (Moura *et al.*, 2003).

Other NOR distribution patterns have been described in Scarabaeidae, as in *Bubas bison* (2n = 20), which has rDNA sites in eight chromosomes. This represents the largest number of NORs observed in this family (Colomba *et al.*, 2006). *Diabroctis mimas* presented NORs in two autosome pairs and in the X chromosome (Bione *et al.*, 2005a).

The NOR distribution patterns suggest that chromosome rearrangements involving the rDNA-bearing chromosomes were important during the evolution of this group. In *D. minas*, for example, it was proposed that a fission in a NOR-bearing autosome followed by its translocation to the X chromosome would explain the presence of rDNA sites in this chromosome (Bione *et al.*, 2005a).

This work presents the first chromosome banding data for the genus *Dichotomius.* The species showed the same reduced diploid number 2n = 18, suggesting that fusions occurred during the chromosome evolution of the group. In spite of the 2n conservation, the different species presented various sex determining mechanisms, variable sizes of CH blocks and of NORs distribution. These data associated with reports from the literature corroborate the occurrence of karyotypic variability in the genus *Dichotomius*.
